# TBX1 Functions as a Tumor Activator in Prostate Cancer by Promoting Ribosome RNA Gene Transcription

**DOI:** 10.3389/fonc.2020.616173

**Published:** 2021-01-26

**Authors:** Jie Cui, Yamin Zhang, Xiaoyue Ren, Lei Jin, Hongyi Zhang

**Affiliations:** ^1^ Department of Oncology, The First Affiliated Hospital, Xi’an Medical University, Xi’an, China; ^2^ School of General Medicine, Xi’an Medical University, Xi’an, China; ^3^ Department of Urology, The First Affiliated Hospital, Xi’an Medical University, Xi’an, China

**Keywords:** prostate cancer, ribosome biogenesis, histone modifications, rRNA, TBX1

## Abstract

TBX1 belongs to an evolutionarily conserved family of transcription factors involved in organ development. TBX1 has been reported to have a hypermethylated cytosine guanine dinucleotide island around its second exon, which was related to prostate cancer (PCa) progression. However, the role and exact mechanism of TBX1 in PCa remains unknown. Using human prostate samples, online data mining and multiple *in vitro* and *in vivo* models, we examined the biological role and underlying mechanisms of TBX1 in PCa. TBX1 was highly expressed in PCa tissues, and high TBX1 expression was positively associated with Gleason score, pathological tumor stage, pathological lymph node stage, extraprostatic extension and disease/progression-free survival. *In vitro* and *in vivo* data demonstrated that TBX1 silencing inhibits PCa cell proliferation and colony formation and increases the cell population at the G0/G1 phase. The exogenous expression of TBX1 rescued these phenotypes. Mechanistically, TBX1 silencing suppressed the expression of 45S ribosomal RNA (rRNA), which was rescued by the exogenous expression of TBX1. TBX1 silencing inhibited the monomethylation of histone 3 lysine 4 (H3K4me1) binding with the non-coding intergenic spacer (IGS) regions of ribosomal DNA (rDNA) and the recruitment of upstream binding factor to the promoter and IGS regions of rDNA. The drug-induced enhancement of H3K4me1 counteracted the effect of TBX1 silencing. These findings indicate that TBX1 exerts its tumor activator function in PCa cells *via* epigenetic control, thereby promoting rRNA gene transcription. Thus, TBX1 may represent a prognostic biomarker and therapeutic target for PCa patients.

## Introduction

Prostate cancer (PCa) is the leading cancer in men and the second leading cause of cancer-related deaths in the United States. The incidence of PCa per 100,000 men in the United States was 123.2 from 2009 to 2013, and the mortality per 100,000 men was 20.0 from 2010 to 2014 (1). The incidence and mortality of PCa in China have also increased considerably in recent years because of the country’s growing aging population. The incidence and mortality of PCa per 100,000 Chinese men peaked at 60.3 and 26.6, respectively, in 2015 (2). Surgery and radiation are generally used to treat localized PCa with curative intent, but some patients develop recurrent or metastatic disease with poor prognosis (3). The current predictors of poor PCa prognosis include Gleason grade, prostate-specific antigen (PSA) and lymph node metastasis (4). Given that biomarkers for prognosis prediction are few, identifying molecular markers for estimating the severity of PCa is necessary. Moreover, understanding the precise molecular mechanisms involved is crucial, and novel molecular targets for PCa treatment should be identified.

T-box (TBX) genes belong to an evolutionarily conserved family of transcription factors involved in organ development. The TBX gene family is divided into five subfamilies, namely, T, TBX1, TBX2, TBX6, and TBR1. TBX genes have essential functions in cell proliferation, differentiation, apoptosis, senescence, invasion and migration, epithelial to mesenchymal transition and several oncogenic pathways (5, 6). Several members of this family, such as TBX2, TBX3, and TBX5, are involved in tumorigenesis (7–9). TBX2 and T-encoded brachyury are associated with PCa progression and aggressiveness (10, 11). TBX1 has been reported to have a hypermethylated cytosine guanine dinucleotide island (CGI) around its second exon in PCa, which was related to disease severity (12). TBX1 rs4819522 polymorphism is associated with a number of pathways with potential influences on serum PSA levels (13). However, knowledge of the role of TBX1 in PCa remains limited.

In the present study, we investigated the clinical impact of TBX1 expression in human PCa samples and the biological role and underlying mechanisms of TBX1 in PCa. We found that TBX1 is overexpressed in primary prostate carcinoma and demonstrated that TBX1 functions as a tumor activator in PCa cells through epigenetic control, thereby increasing rRNA gene transcription.

## Materials and Methods

### Clinical Samples

Primary human prostate tissue specimens were obtained from 280 patients who had undergone radical prostatectomy as a primary treatment from January 2009 to December 2015 at the First Affiliated Hospital of Xi’an Medical University and the Second Affiliated Hospital of Air Forth Medical University. Patients who had undergone preoperative endocrine or radiation therapy were excluded. The samples included 280 primary prostate adenocarcinoma specimens and 120 non-neoplastic specimens. All cases were confirmed by histopathological examination. The area of non-neoplastic tissue adjacent to the tumor (NTAT) was defined by a pathologist. The restrictive criteria included a distance of at least 1.5 mm from tumor cells and exclusion of areas of prostatic intraepithelial neoplasia. If more than one area of NTAT was present, the area farthest from the tumor was chosen (14). Clinicopathological data, including age, serum PSA level, Gleason score, pathological tumor (pT) stage, pathological lymph node (pN) stage, extraprostatic extension and margin status, were retrieved from the files of the pathology departments of the hospitals. Tumor and lymph node status were classified according to the 2010 pTNM classification protocol of the American Joint Committee on Cancer (15), and Gleason scores were graded using the 2005 Gleason grading system (16). Postoperative biochemical recurrence (BCR) was defined as a PSA of 0.4 ng/ml followed by another higher value (17). The Ethics Committees of the First Affiliated Hospital of Xi’an Medical University and the Second Affiliated Hospital of Air Forth Medical University approved the study procedures, and all patients provided written informed consent. All procedures performed in studies involving human participants were conducted in accordance with the ethical standards of the relevant institutional and/or national research committees and the 1964 Helsinki declaration and its later amendments or comparable ethical standards.

### Immunohistochemical Examination

Immunohistochemistry (IHC) was used to detect the expression of TBX1 and ki-67 with anti-TBX1 (BS1876, Bioworld Technology, USA; BS21501R, BIOSS ANTIBODIES, China) and anti-ki67 (BS6667, Bioworld Technology) polyclonal antibodies. Tissue sections (4 µm thick) were deparaffinized and dehydrated. The slides were incubated for 10 min with 3% hydrogen peroxide solution, washed with PBS buffer and then incubated for 15 min with normal goat serum. The slides were subsequently incubated overnight with the primary antibody at 4°C, washed with PBS buffer, incubated for 40 min with the anti-rabbit secondary antibody (BOSTER, Wuhan, China) and stained with diaminobenzidine (BOSTER). The negative control was processed identically but without the primary antibody. All of the slides were assessed independently in a double-blind manner by two experienced pathologists.

### Analysis of TBX1 Expression Using the Oncomine and TCGA Databases

TBX1 mRNA expression and DNA copy numbers were investigated using eight PCa datasets, namely, those of Wallace (18), Singh (19), Grasso (20), Taylor (21), Yu (22), DNA TCGA (23), Nanni (24) and Latulippe (25), from the Oncomine database. TBX1 expression was assessed in PCa and prostate gland tissues. The patients were divided into high and low expression groups according to the log^2^ median intensity values of TBX1 in each study. The relationships between TBX1 expression and clinical parameters were analyzed, and *p* values of <0.05 were considered significant. LinkedOmics (26) was used to analyze the relationship between TBX1 expression and clinical parameters from the Cancer Genome Atlas (TCGA) prostate adenocarcinoma cohort (n = 499) and differentially expressed genes correlated with TBX1. Analysis of Gene Ontology (GO) and KEGG pathways for differentially expressed genes was performed using Gene Set Enrichment Analysis (GSEA). A false discovery rate (FDR) of <0.05 was used as the rank criterion, and 500 simulations were processed. The cBio Cancer Genomics Portal (c-BioPortal; http://cbioportal.org) was used to analyze the relationship between TBX1 expression and prognosis in the TCGA PCa samples.

### Cell Lines and Culture

The human PCa cell lines LNCaP and DU145 were purchased from the Institute of Life Sciences Cell Resource Centre of the Chinese Academy of Sciences (Shanghai, China). The cells were cultured in RPMI 1640 medium supplemented with 10% fetal bovine serum (Invitrogen, USA), penicillin (100 units/ml) and streptomycin (100 μg/ml) in a humidified atmosphere of 5% CO_2_ at 37°C.

### Short Hairpin RNA, Expression Plasmid, and Transfection

A lentiviral vector expressing TBX1-specific short hairpin RNA (shRNA) was obtained from GenePharma (Shanghai, P.R. China). Forward: 5´-CCGGGCGCAGUGGAUGAAGCAAATTCTCGAG UUUGCUUCAUCCACUGCGCTTTTTTTG-3´, Reverse: 5´- AATTCAAAAAGCGCAGUGGAUGAAGCAAATTCTCGAG UUUGCUUCAUCCACUGCGCTT-3´. shRNA lentiviral control vectors (Forward:5´-UUCUCCGAACGUGUCACGUTT -3´, Reverse: 5´- ACGUGACACGUUCGGAGAATT -3´) were also used. Cells were transfected using X-tremeGENE HP DNA Transfection Reagent (Roche, Mannheim, Germany) as recommended by the manufacturer. After transfection, cells displaying stable endogenous TBX1 silencing were obtained after treatment with 1.0 μg/ml puromycin (Sigma–Aldrich). A TBX1 expression plasmid (pcDNA3.1/myc-His(−) A -TBX1) containing a Myc tag was obtained from Yingrun Biotechnology (Changsha, P.R. China). pcDNA3.1/myc-His(−) A, an empty vector, was also used. TBX1-silenced cells were transfected with the TBX1 expression plasmid or empty vector. After 48 h, the cells were collected for further experiments.

### Quantitative Real-Time Polymerase Chain Reaction

Quantitative real-time polymerase chain reaction (qPCR) was used to investigate TBX1 and 45S ribosome RNA (rRNA) levels. PCa cells were treated with shControl, shTBX1, shTBX1 combined with the TBX1 expression plasmid, shTBX1 combined with the empty vector or shTBX1 combined with tranylcypromine (TCP). Total RNA was extracted using Trizol (Invitrogen) according to the manufacturers’ protocol. The cDNA was synthesized using the PrimeScript RT reagent kit (Takara, Dalian, China), and mRNA levels were analyzed using an ABI 7500HT real-time PCR system (Applied Biosystems, USA). The 2^-ΔΔCT^ method was used to analyze the data. Each sample was run in triplicate, and GAPDH was used as the internal control. The primers used are presented in **Supplementary Table 1**.

### Western Blot Analysis

Cellular total protein was extracted with radioimmunoprecipitation assay buffer containing protease inhibitors. For histone extraction, cells were collected by refrigerated centrifugation at 300 × *g* for 10 min. The pellet was incubated for 30 min in hypotonic lysis buffer [10 mM Tris-Cl (pH 8.0), 1 mM KCl, 1.5 mM MgCl_2_, 1 mM DTT and protease inhibitors]. The nuclei were resuspended in acid-extraction buffer (0.4 N H_2_SO_4_), and the extracted histones were precipitated with trichloroacetic acid and resuspended in deionized water. Equal amounts of protein lysates were separated by sodium dodecyl sulfate–polyacrylamide gel electrophoresis and then transferred onto polyvinylidene fluoride membranes (Millipore). The membranes were incubated with primary antibodies, including anti-TBX1 (ab109313, Abcam), anti-H3K4me1 (ab176877, Abcam), anti-H3K4me2 (ab32356, Abcam), anti-H3K4me3 (ab8580, Abcam), anti-histone H3 (ab176842, Abcam) and anti-β-actin (BS6007MH, Bioworld Technology), overnight at 4°C and then with horseradish peroxidase-conjugated secondary antibodies (Sinopept) for 1 h at room temperature. Visualization was performed using an enhanced chemiluminescence detection system (Millipore). Experiments were repeated in triplicate.

### Cell Viability and Colony Formation Assays

Cell Counting Kit-8 (CCK-8) and plate colony formation assays were used to investigate cell viability and proliferation. For the CCK-8 assays, 1,000–2,000 cells per well were placed on 96-well plates. After treatment, CCK-8 solution (20 μl/well) was added to each well, and the plates were incubated for 4 h at 37°C. The absorbance of each well was measured at 450 nm with a microplate reader to assess cell viability. For the colony formation assays, 500–1,000 cells (of the various cell lines) were seeded into the wells of six-well plates. The medium was refreshed every 3 days. After 14 days of culture, the colonies that had formed (≥100 cells per colony) were fixed with 4% paraformaldehyde, stained with 1.25% crystal violet and manually counted. The experiments were performed in triplicate.

### Cell Cycle Assay

The effects of TBX1 on the cell cycle were determined by flow cytometry. Briefly, the prostate cancer cells were collected, fixed with 75% ethanol, stored overnight at −20°C and then resuspended in PBS. Propidium iodide (30 μg/ml) and RNase (40 μg/ml) were added to the cells, and the resulting solution was incubated for 30 min in the dark. Cell cycle distributions were analyzed by flow cytometry (FACSCalibur, BD Biosciences, San Jose, CA, USA).

### ChIP–qPCR

Chromatin immunoprecipitation (ChIP) assays were performed using a simple ChIP™ enzymatic IP kit (Cell Signalling Technology) according to the manufacturer’s protocol. Anti-H3K4me1 (ab176877, Abcam) and anti-upstream binding factor (UBF; sc13125, Santa Cruz) antibodies were used to immunoprecipitate the cross-linked chromatin. Non-specific mouse IgG and histone H3 were used as negative and positive controls, respectively. The immunoprecipitated DNA was amplified using specific primers to amplify multiple regions of the rRNA genes. DNA amplification was quantified by qPCR analysis using SYBR Green PCR master mix (ABI, MA, USA) on an ABI 7500 real-time PCR system. The percentage of DNA brought down by ChIP (percent input) was calculated. Experiments were repeated in triplicate, and the primers used are presented in **Supplementary Table 2**.

### Animal Studies

Male BALB/c nude mice aged 6 weeks were obtained from Silaike Experimental Animal Company (Shanghai, China). The Ethics Committee of the First Affiliated Hospital of Xi’an Medical University approved the experiment protocols. The study was carried out in accordance with the National Institutes of Health guidelines for the care and use of laboratory animals (NIH Publication No. 8023, revised 1978). The mice were divided into two groups (six mice per group). In one group, 1×10^7^ TBX1-silenced DU145 cells were injected subcutaneously into the right back flank of each mouse. In the other group, control cells were injected. The volumes of the implanted tumors were measured every other day. All of the mice were euthanized on day 15, and the resulting tumors were excised and weighed. Tumors from representative mice were embedded in paraffin and sectioned into slices measuring 4 µm thick. IHC was used to investigate the expression of TBX1 and ki-67.

### Statistical Analysis

Categorical variables were analyzed using the *χ* ^2^ test. Continuous variables were analyzed using independent sample *t*-tests. The Kaplan–Meier method and log-rank test were used to determine differences in BCR-free survival, and Cox regression analysis was used to identify independent risk factors. All of the data were analyzed using SPSS version 19.0 (SPSS, Inc., Chicago, IL, USA), and a *p* value of <0.05 was considered a statistically significant difference.

## Results

### TBX1 Is Frequently Overexpressed in PCa Tissues

**Figure 1:**
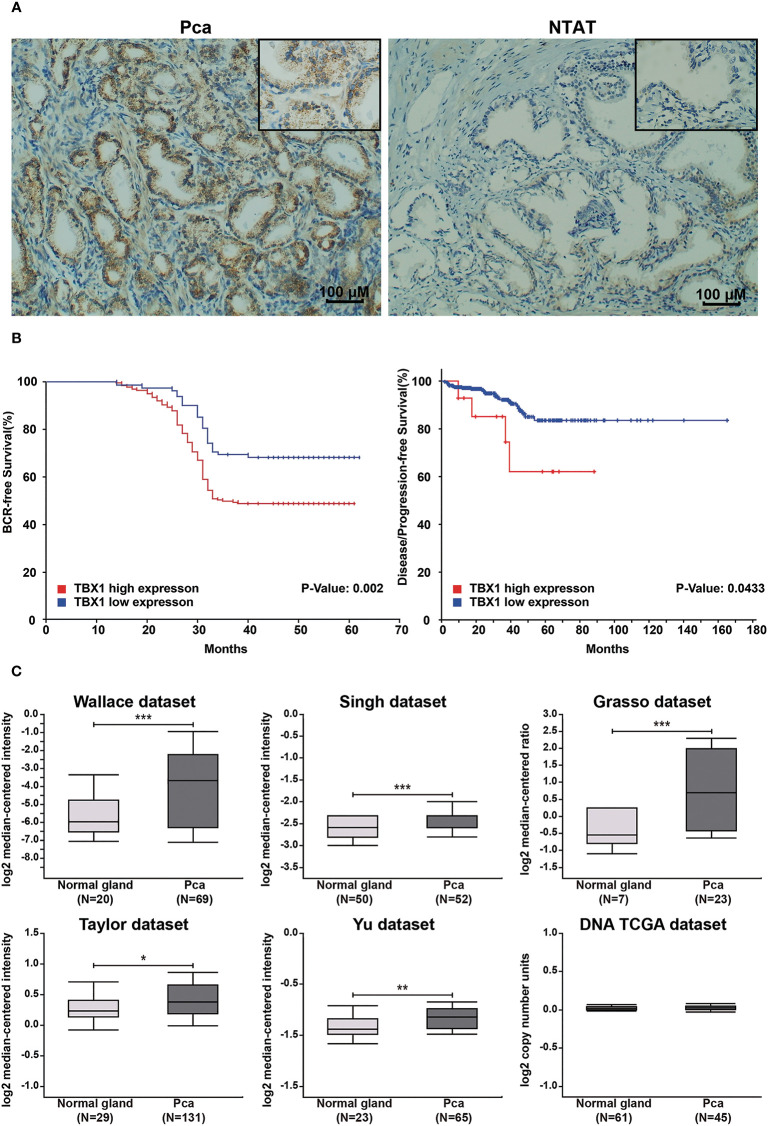
High TBX1 expression is related to poor prognosis in PCa. **(A)** Left, TBX1 is expressed at high levels in PCa tissues, and TBX1-positive cells exhibit nuclear and cytoplasm staining. Right, TBX1 is absent in non-neoplastic tissues. **(B)** Kaplan–Meier curves demonstrating the survival distribution stratified by TBX1 expression level. Left, biochemical recurrence-free survival determined from 280 PCa samples in the study. Right, disease/progression-free survival determined from 499 PCa samples in the TCGA database. **(C)** Analysis of microarray TBX1 expression data from the Oncomine database. Analysis of the log^2^ median intensity values of TBX1 was conducted using six different datasets. TBX1 is commonly expressed at high levels in PCa tissues. *p < 0.05; **p < 0.01; ***p < 0.001. Prostate cancer, PCa; Non-neoplastic tissue adjacent to the tumor, NTAT; Biochemical recurrence (BCR).

TBX1 protein expression in 280 primary prostate carcinoma tissues and 120 normal prostate gland tissues was assessed using IHC. TBX1 was primarily expressed in the nuclei and cytoplasm of the epithelial cells. As shown in **Figure 1A**, PCa tissues presented higher levels of TBX1 staining compared with normal prostate gland tissues. TBX1 overexpression was found in 198/280 (70.7%) of the PCa tissues and 24/120 (20%) of the normal prostate tissues (*p* < 0.01). We assessed the clinical impact of TBX1 expression in 280 PCa patients, and results showed that high TBX1 expression is positively associated with Gleason score (*p* = 0.018) and pT stage (*p* = 0.027; **Table 1**). Cox regression analysis indicated that TBX1 expression is independently associated with shorter BCR-free survival (*p* = 0.01; **Table 2**). A significant difference in time to BCR was found between the high- and low-TBX1 expression groups (log-rank *p* = 0.002; **Figure 1B**, left panel).

**Table 1:** Correlation of TBX1 expression with clinicopathologic parameters in 280 PCa patients.

Clinical parameters	TBX1		
	n	High expression (%)	*p*
Age, y			
<60	42	29(69.0)	0.854
≥60	238	169(71)	
Gleason score			
<3+4	205	137(66.8)	**0.018**
≥4+3	75	61(81.3)	
pT stage			
pT1-2	218	147(67.4)	**0.027**
pT3-4	62	51(82.3)	
pN stage			
N0	262	185(70.6)	1.000
N1	18	13(72.2)	
Extracapsular extension			
No	235	162(68.9)	0.155
Yes	45	36(80.0)	
Surgical margins			
No	238	165(69.3)	0.272
Yes	42	33(78.6)	
PSA			
<20 ng/ml	140	102(72.9)	0.512
≥20 ng/ml	140	96(68.6)	

The bold values represent p < 0.05.

**Table 2:** Association of various parameters with BCR-free survival in 280 patients with PCa on univariate and multivariate analyses.

Variables	Univariate analysis		Multivariate analysis	
	HR (95%CI)	*p* value	HR (95%CI)	*p* value
TBX1expression	1.94 (1.26-3.00)	0.003	0.54 (0.35–0.84)	**0.01**
Age	1.36(0.81-2.30)	0.25	1.08 (0.63–1.84)	0.80
Gleason score	3.16 (2.21-4.51)	<0.001	1.85 (1.14–3.01)	**0.01**
pT stage	2.65 (1.82-3.85)	<0.001	0.51 (0.29–0.90)	**0.02**
pN stage	2.15 (1.18-3.89)	0.01	0.82 (0.43–1.58)	0.56
Extracapsular extension	0.27(0.18-0.40)	<0.001	0.33 (0.21–0.51)	**<0.001**
Surgical margins	4.61(3.10-6.87)	<0.001	5.96 (3.34–10.64)	**<0.001**
PSA	1.40 (0.98-1.97)	0.07	1.53 (1.07–2.21)	**0.02**

BCR, Biochemical recurrence; PCa, Prostate cancer. The bold values represent p<0.05.

### Genomic Alterations of TBX1 in PCa From the Oncomine and TCGA Databases

TBX1 transcription levels in multiple PCa studies from Oncomine (i.e. Wallace, Singh, Grasso, Taylor, Yu, DNA TCGA) were evaluated; the dataset comprised 385 primary PCa samples and 190 normal prostate gland samples. The results showed that the mRNA expression of TBX1 is significantly higher in PCa tissues than in normal prostate gland tissues. The fold changes of TBX1 ranged from 1 to 4 and ranked within the top 25% in terms of mRNA expression. DNA copy numbers were similar between PCa and normal prostate tissues (**Figure 1C**). The microarray datasets of Oncomine, which includes five studies of localized PCa with clinical data (i.e. Taylor, Yu, Wallace, Nanni, Latulippe), were explored to analyze the relation between TBX1 mRNA level and clinical parameters. The results showed that TBX1 mRNA level is related to Gleason score in the Taylor (*p* = 0.03) and Latulippe (*p* = 0.035) studies, pT stage in the Taylor (*p* = 0.012) and Nanni (*p* = 0.076) studies, N stage in the Taylor study (*p* = 0.001) and extraprostatic extension in the Wallace study (*p* = 0.03; **Table 3**). Next, we analyzed the clinical–pathological parameters of 499 PCa samples in the TCGA database using LinkedOmics and c-BioPortal. TBX1 mRNA level was significantly related to pT stage (*p* = 0.021), pN stage (*p* = 0.008) and disease/progression-free survival (*p* = 0.043; **Figure 1B**, right panel). These data suggest that high TBX1 expression is associated with poor prognosis in PCa patients.

**Table 3:** Correlation of TBX1 microarray expression profiles with clinicopathologic parameters in different datasets from the oncomine database.

Clinical parameters	Taylor			Yu			Wallace			Nanni			Latulippe		
	n	High (%)	p	n	High (%)	p	n	High (%)	p	n	High (%)	p	n	High (%)	p
Age, y															
<61	98	48(49.0)	0.731	NA	NA	NA	NA	NA	NA	9	3(33.3)	0.193	9	4(44.4)	0.795
≥61	52	27(51.9)		NA	NA	NA	NA	NA	NA	13	8(61.5)		14	7(50.0)	
Gleason score															
<7	41	22(53.7)	**0.03**	18	9(50)	0.851	18	10(55.6)	0.732	4	1(25.0)	0.357	2	0(0)	**0.035**
7	76	30(39.5)		27	15(55.6)		48	23(47.9)		17	9(52.9)		14	5(35.7)	
≥7	22	16(72.7)		19	9(47.4)		3	2(66.7)		1	1(100)		7	6(85.7)	
pT stage															
pT2	86	33(38.4)	**0.012**	25	10(40.0)	0.322	38	17(44.7)	0.272	NA	NA	0.076	10	3(30.0)	0.161
pT3	47	31(66.0)		37	22(59.5)		30	18(60.0)		14	5(37.5)		11	6(54.5)	
pT4	8	5(62.5)		2	1(50.0)		1	0(0)		8	6(75.0)		2	2(100)	
N stage															
N0	105	42(40.0)	**0.001**	58	32(55.2)	NA	NA	NA	NA	NA	NA	NA	19	9(47.4)	0.924
N1	16	13(81.3)		5	0(0)		NA	NA	NA	NA	NA	NA	4	2(50.0)	
Extracapsular extension															
No	NA	NA	NA	NA	NA	NA	44	18(40.9)	**0.03**	NA	NA	NA	NA	NA	NA
Yes	NA	NA	NA	NA	NA	NA	25	17(68.0)		NA	NA	NA	NA	NA	NA

The bold values represent p<0.05.

### GO and KEGG Pathway Analysis of TBX1 Co-Expression Genes in PCa

**Figure 2:**
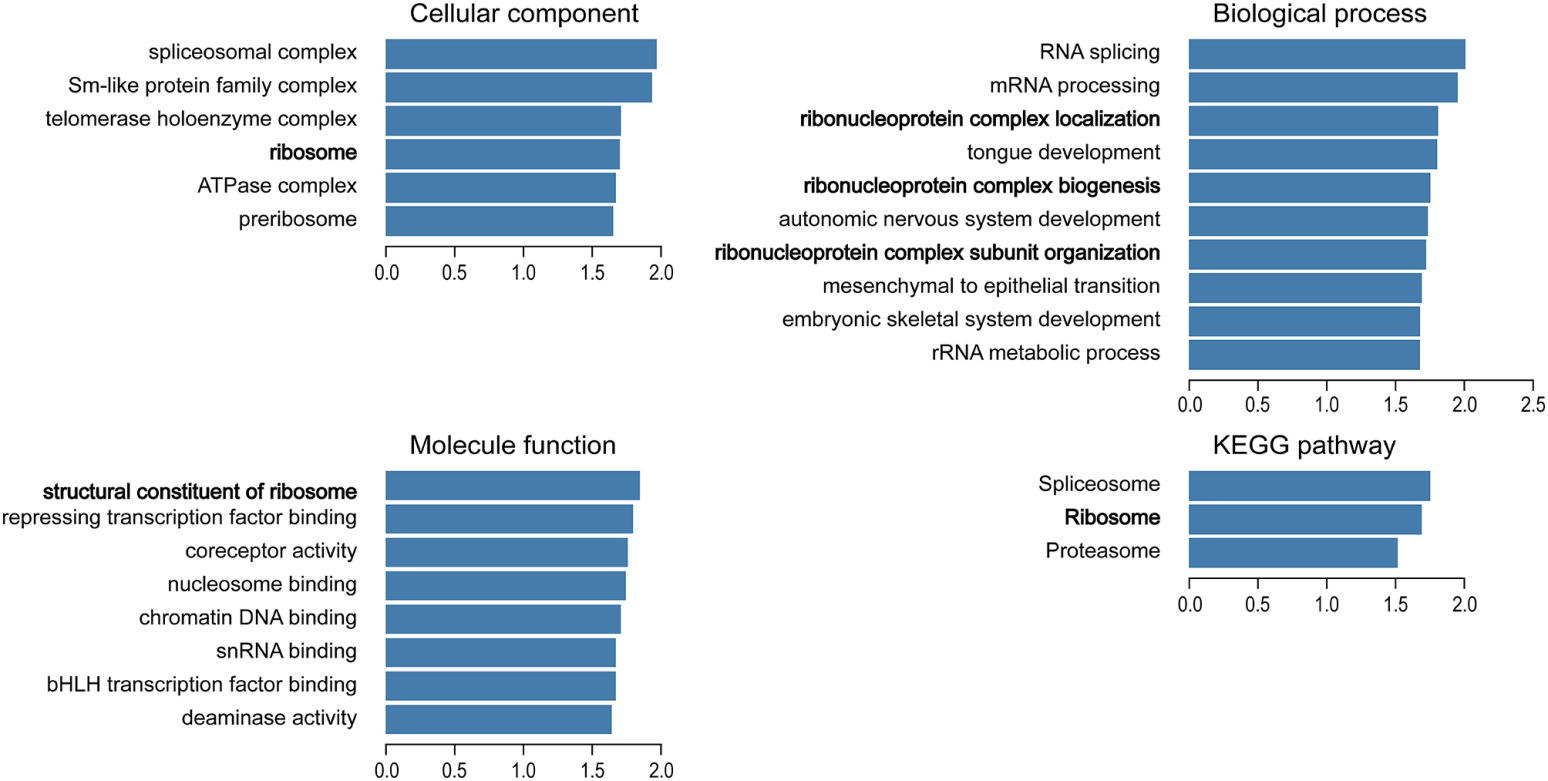
Analysis of significantly enriched GO annotations and KEGG pathways of differentially expressed genes correlated with TBX1 from PCa patients in the TCGA database using GSEA. The blue column represents the normalized enrichment scores. FDR < 0.05. Gene Ontology, GO; Gene Set Enrichment Analysis, GSEA.

We used the Function module of LinkedOmics to analyze the mRNA sequencing data of 499 patients with prostate adenocarcinoma in the TCGA database. A total of 2,596 genes (dark red dots) were positively related to TBX1, whereas 3,050 genes (dark green dots) were negatively related to TBX1 (FDR < 0.01) in the volcano plot (**Supplemental Figure 1**). GO term and KEGG pathways analysis by GSEA showed that the differentially expressed genes correlated with TBX1 are mainly ribosome-related genes which involved in the biological processes of ribonucleoprotein complex localization, biogenesis and structural constituent of ribosome. Analysis of KEGG signaling pathways revealed gene enrichment in ribosomes (**Figure 2**). These results suggest that TBX1 may function in the core of the ribosome.

### TBX1 Promotes PCa Cell Growth *In Vitro* and *In Vivo*

**Figure 3:**
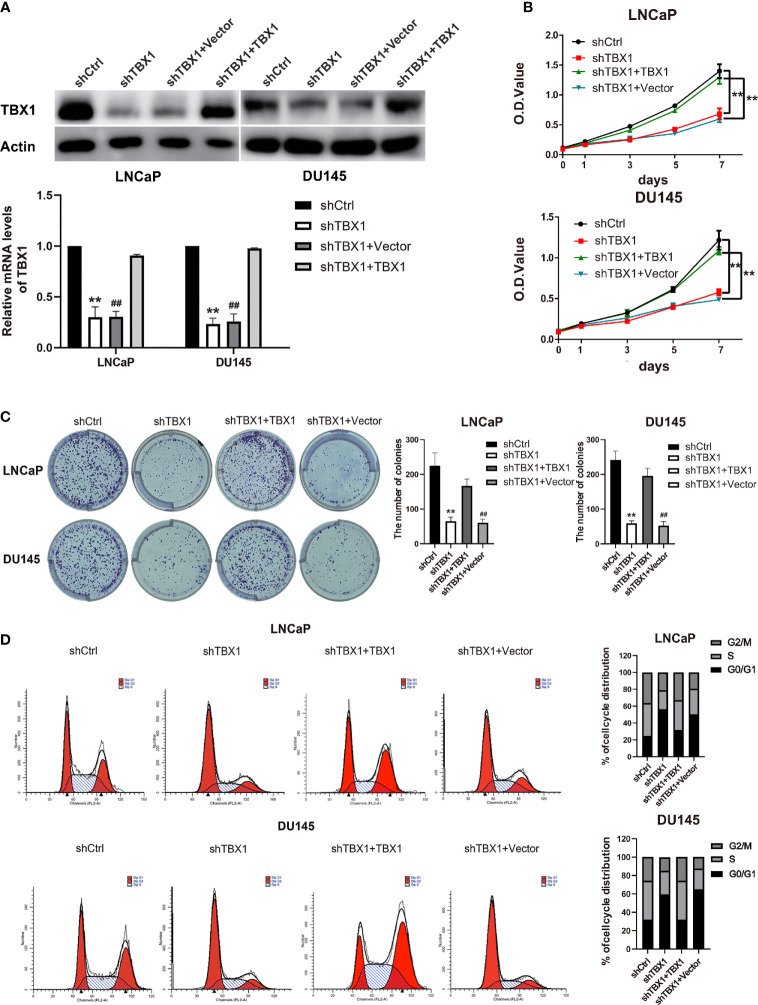
TBX1 promotes PCa cell growth *in vitro*. **(A)** Western blot analysis (upper panel) and qPCR (lower panel) assay results showing that shTBX1 transfection dramatically decreases TBX1 expression and that the TBX1 expression plasmid increases TBX1 expression in LNCaP and DU145 cells. The data represent the mean ± SD of three independent experiments. **(B)** TBX1 silencing dramatically inhibits PCa cell viability, which is rescued by the exogenous expression of TBX1. The data represent the mean ± SD of three independent experiments. **(C)** TBX1 silencing significantly inhibits the colony formation of PCa cells, which is rescued by the exogenous expression of TBX1. Left panel, representative images of colony formation. Right panel, quantitative analysis of colon numbers. The data represent as the mean ± SD of three independent experiments. **(D)** TBX1 silencing increases the percentage of cells in the G0/G1 phase. Cell cycle distributions were investigated by flow cytometry. The data represent the mean ± SD of three independent experiments. **, *p* < 0.01, shTBX1 vs. shCtrl/shTBX1+TBX1; ^##^, *p* < 0.01, shTBX1+Vector vs. shCtrl/shTBX1+TBX1.

**Figure 4:**
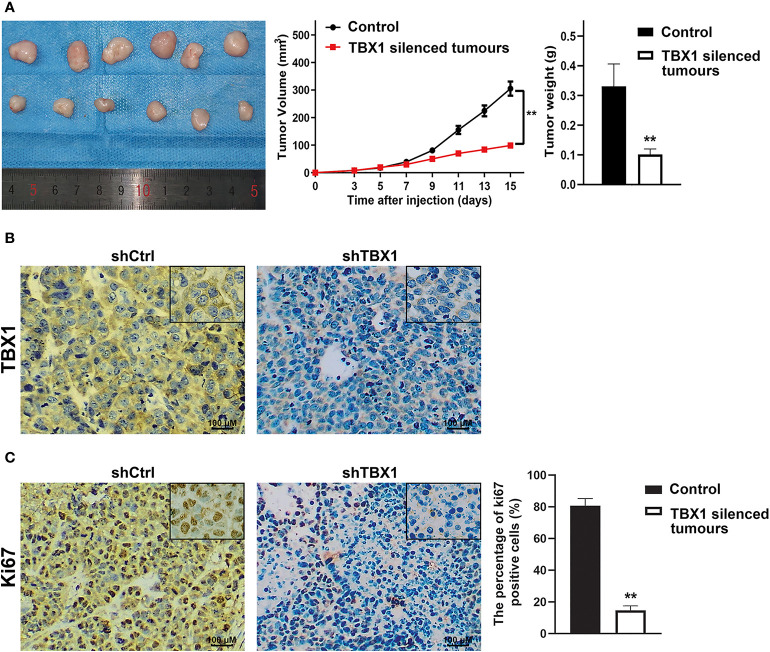
TBX1 silencing decreases the growth of implanted PCa cells tumors *in vivo* **(A)** Left panel, photographs of tumors implanted with TBX1-silenced DU145 cells and control tumors from nude mice. Middle panel, tumor growth curves of the TBX1-silenced and control groups. Right panel, histogram of mean tumor weights. The data are shown as mean ± SD (n = 6/group). **(B)** Representative TBX1 staining of xenograft tumors obtained from the TBX1-silenced and control groups. **(C)** Left panel, representative Ki-67 staining of xenograft tumors obtained from the TBX1-silenced and control groups. Right panel, histogram representing the percentage of Ki-67-positive cells from five microscopic fields of the TBX1-silenced and control groups. The data are shown as mean ± SD (n = 6/group). **p < 0.01, shTBX1 silencing tumors vs control.

To investigate the effect of TBX1 on cell viability and proliferation, we silenced TBX1 expression with shRNA-TBX1 (shTBX1) in LNCaP and DU145 cells (**Figure 3A**). CCK-8 assays revealed a significant decrease in the viability of shTBX1-transfected cells compared with that of control cells (**Figure 3B**). TBX1-silenced cells were transfected with the TBX1 expression plasmid (**Figure 3A**), and we found that the exogenous expression of TBX1 rescues the decrease in cell viability due to TBX1 silencing (**Figure 3B**). Colony formation assays revealed a significant decrease in the number of colonies formed in shTBX1-transfected cells compared with that in control cells; similarly, the exogenous expression of TBX1 rescued the decrease in number of colonies due to TBX1 silencing (**Figure 3C**). Flow cytometry was used to investigate the effect of TBX1 on the cell cycle, and results revealed a significant increase in the percentage of cells in the G0/G1 phase in shTBX1-transfected cells compared with that in control cells. The percentage of G0/G1 phase cells increased from 24.6% ± 11.2% to 56.2% ± 18.5% in LNCaP cells (*p <* 0.05) and from 32.0% ± 13.7% to 59.4% ± 22.8% in DU145 cells (*p <* 0.05). The exogenous expression of TBX1 rescued the increased percentage of G0/G1 phase cells due to TBX1 silencing (**Figure 3D**). We examined the effect of TBX1 silencing on the growth of implanted DU145 cells tumors *in vivo* and found significant decreases in the tumor volumes and weights of TBX1-silenced cells compared with those in control cells (**Figure 4A**). The percentage of Ki-67-positive cells also significantly decreased in tumors with stable TBX1 silencing compared with that in control tumors (**Figures 4B, C**). These results indicate that TBX1 is a tumor activator in PCa.

### TBX1 Promotes Ribosome RNA Expression

**Figure 5:**
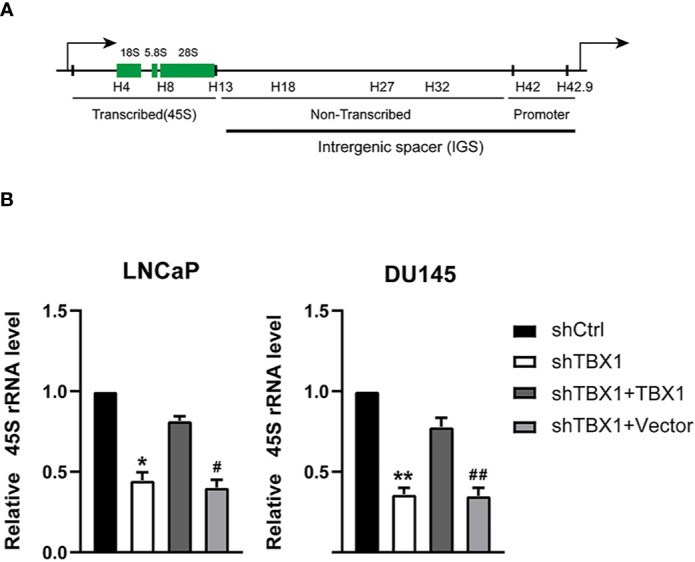
TBX1 silencing suppresses the expression of 45S rRNA, which is rescued by the exogenous expression of TBX1. **(A)** Schematic representation of one human rDNA repeat. **(B)** qPCR results of the effect of TBX1 on the expression of 45S rRNA in LNCaP and DU145 cells. The data represent the mean ± SD of three independent experiments. **p < 0.01; *p < 0.05, shTBX1 vs. shCtrl/shTBX1+TBX1; ^##^ *p* < 0.01; ^#^ *p* < 0.05, shTBX1+Vector vs. shCtrl/shTBX1+TBX1.

We have demonstrated TBX1 may function in the core of the ribosome. Ribosome biogenesis begins with the transcription of ribosomal RNA genes. Thus, we tested the effect of TBX1 on the expression of 45S rRNA through qPCR. TBX1 silencing dramatically decreased the expression of 45S rRNA in LNCaP and DU145 cells. When TBX1-silenced cells were transfected with the TBX1 expression plasmid, the exogenous expression of TBX1 rescued the decreased expression of 45S rRNA due to TBX1 silencing (**Figure 5**).

### TBX1 Silencing Decreases the Enrichment of H3K4me1 and UBF in rRNA Genes

**Figure 6:**
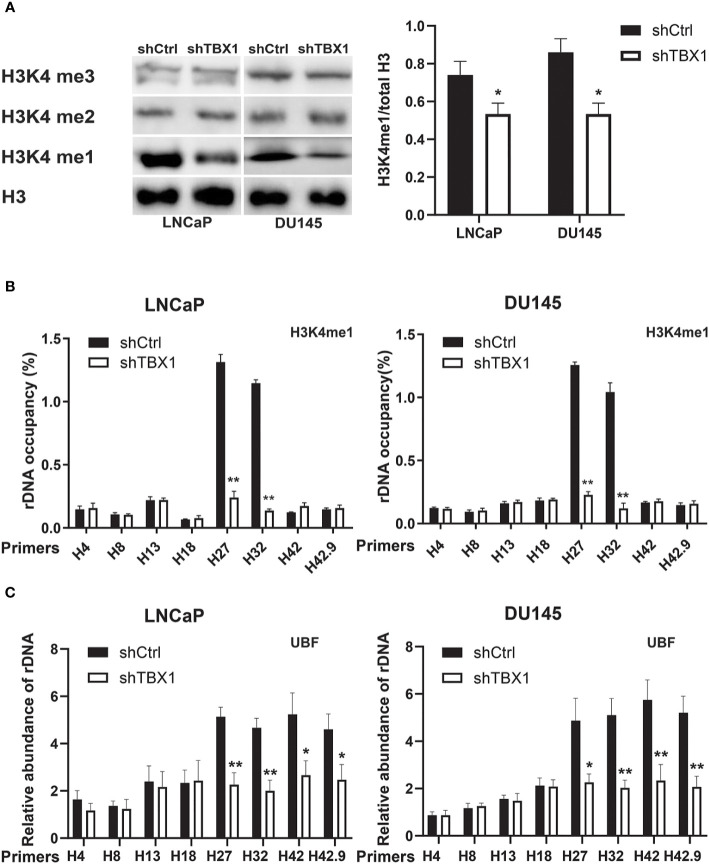
TBX1 silencing inhibits H3K4me1 and UBF binding with rRNA genes. **(A)** Left, Western blot analysis results of H3K4me1 expression. Right, quantitative results of the left panel. **(B)** CHIP–qPCR analysis results of the level of H3K4me1 binding with rRNA genes. **(C)** CHIP–qPCR analysis results of the level of UBF binding with rRNA genes. The data represent the mean ± SD of three independent experiments. **p < 0.01; *p< 0.05, shTBX1 vs. shCtrl. Upstream binding factor, UBF.

Given that H3K4me1 enrichment in rRNA genes increases ribosomal DNA (rDNA) transcription by enhancing the recruitment of UBF, the key transcription regulator of rRNA synthesis, to rRNA genes (27), we investigated the role of TBX1 in H3K4me1 expression by Western blotting. TBX1 silencing caused a reduction in H3K4me1 levels but had no effect on H3K4me2 and H3K4me3 expression (**Figure 6A**). H3K4me1 enrichment in rRNA genes was determined by CHIP–qPCR. Enrichment of H3K4me1 occurred in the non-coding intergenic spacer (IGS) region (primers H27 and H32) and decreased following TBX1 silencing (**Figure 6B**). We examined the recruitment of UBF to rRNA genes and found that TBX1 silencing decreases the recruitment of UBF to the rDNA promoter and IGS region (**Figure 6C**).

### H3K4me1 Enhancement Partially Counteracts the Effect of TBX1 Silencing

**Figure 7:**
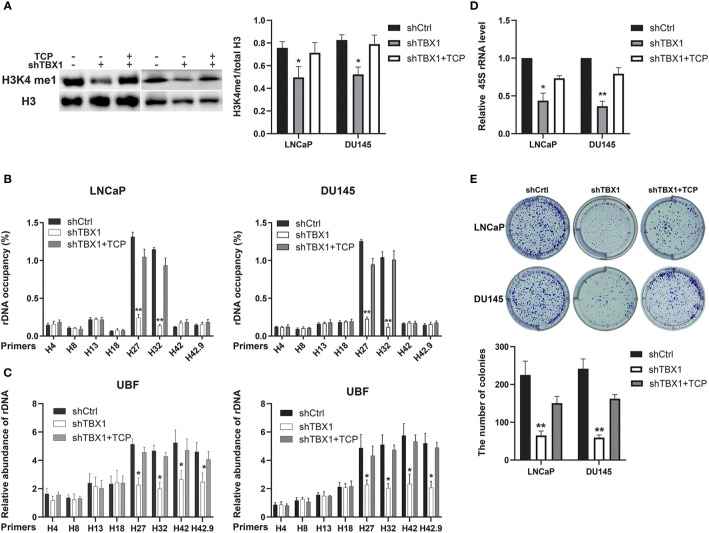
H3K4me1 enhancement partially counteracts the effect of TBX1 silencing. **(A)** Left, Western blot results showing that TCP treatment partially rescues the decrease in H3K4me1 due to TBX1 silencing. Right, quantitative results of the left panel. **(B)** CHIP–qPCR analysis results showing that TCP treatment partially rescues the observed decrease in level of H3K4me1 binding with rRNA genes due to TBX1 silencing. **(C)** CHIP–qPCR analysis results showing that TCP treatment partially rescues the decrease in level of UBF binding with rRNA genes due to TBX1 silencing. **(D)** qPCR analysis results showing that TCP treatment partially rescues the decrease in 45S rRNA due to TBX1 silencing. **(E)** TCP treatment partially rescues the decrease in colony formation due to TBX1 silencing. Upper panel, representative images of colony formation. Lower panel, quantitative analysis of colon numbers. The data represent the mean ± SD of three independent experiments. *p < 0.05; **p < 0.01, shTBX1 vs. shTBX1+TCP/shCtrl. Tranylcypromine, TCP; upstream binding factor, UBF.

We investigated whether the drug-induced enhancement of H3K4me1 could counteract the effect of TBX1 silencing. TCP, an inhibitor of Lsd1/2 histone demethylases (28), was used in this study. TCP treatment rescued the observed decreases in H3K4me1 level (**Figure 7A**), H3K4me1 binding with rRNA genes (**Figure 7B**), UBF binding with rRNA genes (**Figure 7C**) and expression of 45S rRNA due to TBX1 silencing (**Figure 7D**). We also evaluated the effect of TCP treatment on cell proliferation and found that TCP treatment dramatically increases the number of colonies in TBX1-silenced cells compared with that in the control group (**Figure 7E**). These findings indicate that H3K4me1 enhancement partially counteracts the effects of TBX1 silencing.

## Discussion

The transcription factor TBX1 is involved in cell morphology, dynamics, interaction, adhesion, proliferation and differentiation and lymphatic vessel development (29–31). Although TBXT and TBX2, which are other members of the T-box family, are understood to play important roles in PCa aggressiveness, the biological roles of TBX1 in prostate tumorigenesis and progressiveness remain unknown. In this study, our data provide strong evidence supporting the tumor-promoting role of TBX1 in PCa.

We found that TBX1 is aberrantly upregulated in PCa tissues relative to normal prostate tissues by immunostaining. High TBX1 expression was positively associated with Gleason score, T stage, N stage, extraprostatic extension and poor prognosis of PCa. TBX1 was primarily expressed in the nucleus and cytoplasm. These observations indicate that TBX1 regulates cell behavior in transcriptional and non-transcriptional ways. Our findings are corroborated by the results of bioinformatics analysis of public sequencing data. Data from the Oncomine and TCGA databases confirmed that TBX1 mRNA levels in PCa are significantly increased relative to those in normal prostate tissues but that DNA copy numbers are similar between samples. High TBX1 levels were associated with poor outcomes. TCGA data were analyzed using the LinkedOmics database because association and functional enrichment analyses are widely used and well understood in biomedical research (26). TBX1 gene has been reported to be hypermethylated in breast cancer (32, 33) and PCa (12). The hypermethylation at CGIs was associated with a late stage and lethal neuroendocrine phenotype of PCa (12). DNA copy numbers did not remarkably change in PCa samples from TCGA database. Thus, we speculate that TBX1 overexpression in PCa may result from alterations at the transcriptional level and be related to epigenetic modifications. TBX1 overexpression may participate in tumorigenesis and cancer progression and be a potential molecular marker of PCa.

We demonstrated that TBX1 silencing inhibits PCa cell viability and proliferation and induces G0/G1 phase arrest; these effects are rescued by the exogenous expression of TBX1. Some studies have found that TBX1 plays a positive role in the cell cycle. Verdelli demonstrated that TBX1 silencing in HEK293 cells and parathyroid adenoma-derived cells increases the proportion of cells in the G0/G1 phase (34). Chen found that TBX1-deficient hair follicle stem cells become progressively depleted and remain quiescent for longer times than normal, although they eventually cycle (35). TBX1 also functions as a potential tumor suppressor. TBX1 is frequently inactivated by promoter methylation and inhibits thyroid cancer growth by inhibiting the PI3K/AKT and MAPK/ERK signaling pathways (36). Therefore, the role of TBX1 in cancer may be tissue specific or cell-type dependent.

TBX1 has many important physiological functions. Analysis of differentially expressed genes in the TCGA database revealed that the TBX1 co-expressed genes are mainly involved in the biogenesis of ribonucleoprotein complex. Ribosome biogenesis promotes cell cycle progression and is upregulated in cancer cells to meet cell demands. Cell growth regulation ultimately depends on the control of new ribosome synthesis. rDNA transcription is the major rate-limiting step of ribosome biogenesis and essential for cell growth (37–39). Therefore, we investigated the role of TBX1 in rDNA transcription. We found that TBX1 promotes the expression of 45S rRNA in PCa cells. H3K4me1 is a histone modification mark of enhancer (40) and enriches at the DNA double-strand break regions of rRNA genes; it contributes to rDNA transcription by facilitating the recruitment of UBF to rDNA (27). TBX1 regulates region-specific H3K4me1 enrichment by interacting with and recruiting histone methyltransferases (41). Thus, we investigated the effect of TBX1 on H3K4me1 enrichment in rDNA and the recruitment of UBF to rDNA in PCa cells. TBX1 silencing caused significant reductions in the enrichment of H3K4me1 at the IGS regions of rRNA genes, and decreased the recruitment of UBF to the rDNA promoter and IGS region. Hence, we suggest that TBX1 promotes H3K4me1 enrichment at the IGS regions of rDNA, which increases the recruitment of UBF to the promoter and IGS regions of rDNA, the transcription of which subsequently increases in PCa.

We further investigated whether drug-induced H3K4me1 enhancement counteracts the effect of TBX1 silencing by using TCP, an inhibitor of Lsd1/2 histone demethylases. TCP treatment significantly increased the expression of H3K4me1 and rescued the observed decreases in H3K4me1 binding with rDNA, UBF binding with rDNA, 45S rRNA gene transcription and cell viability caused by TBX1 silencing. These findings support the view that the regulation of H3K4me1 enrichment in rRNA genes is important to the biological role of TBX1 in PCa.

In this study, we found that TBX1 is primarily expressed in the nuclei and cytoplasm of epithelial cells. The role of TBX1 in the cell cytoplasm has yet to be elucidated. Wnt-β-catenin signaling is a crucial upstream regulator of TBX1- fibroblast growth factor (FGF) 8 signaling pathway (42). TBX1 interacts with Pax2 protein, which may regulate TBX1 expression during renal development by directly binding with the promoter area (43). Thus, we speculate that TBX1 in the cytoplasm regulates cell behavior by interacting with other proteins in a non-transcriptional manner.

In conclusion, the present work provides multilevel evidence of the overexpression of TBX1 in PCa, with clinicopathological and prognostic significance. Mechanistically, TBX1 promotes PCa growth through epigenetic control, thereby increasing rRNA gene transcription. TBX1 may represent a prognostic biomarker and therapeutic target for patients with PCa.

## Data Availability Statement

The datasets presented in this study can be found in online repositories. The names of the repository/repositories and accession number(s) can be found in the article/**Supplementary Material**.

## Ethics Statement

The studies involving human participants were reviewed and approved by The Ethics Committees of the First Affiliated Hospital of Xi’an Medical University. The patients/participants provided their written informed consent to participate in this study. The animal study was reviewed and approved by The Ethics Committees of the First Affiliated Hospital of Xi’an Medical University. Written informed consent was obtained from the individual(s) for the publication of any potentially identifiable images or data included in this article.

## Author Contributions

JC: Conceptualization, data curation, formal analysis, funding acquisition, and writing original draft. YZ: Acquisition of data, formal analysis, software, and editing of the manuscript. XR: Acquisition of data, formal analysis, software, and editing of the manuscript. LJ: Analysis and interpretation of data. HZ: Supervision, project administration, and critical revision of the manuscript. All authors contributed to the article and approved the submitted version.

## Funding

The study was supported by Science and Technology Department of Shaanxi Province (Grant No. 2018SF-234).

## Conflict of Interest

The authors declare that the research was conducted in the absence of any commercial or financial relationships that could be construed as a potential conflict of interest.

## Supplementary Material

Supplemental Figure 1 Genes differentially expressed in correlation with TBX1 in PCa (LinkedOmics). Left, the correlations between TBX1 and genes differentially expressed was analyzed using the Pearson test; Middle, the TOP 50 genes positively correlated with TBX1 were shown using the heat map; Right, the TOP 50 genes negatively correlated with TBX1 were shown using the heat map. The red color represents positively correlated genes and the green color represents negatively correlated genes. Prostate cancer, Pca.

## Abbreviations

PCa, prostate cancer; PSA, prostate-specific antigen; T-box, TBX; EMT, epithelial to mesenchymal transition; pT, pathological tumor; pN, pathological lymph node; BCR, biochemical recurrence; TCGA, the Cancer Genome Atlas; GO, Gene Ontology; GSEA, Gene Set Enrichment Analysis; FDR, false discovery rate; c-BioPortal, cBio Cancer Genomics Portal; rRNA, ribosomal RNA; rDNA, ribosomal DNA; H3K4me1, monomethylation of histone 3 lysine 4; H3K4me2, di-methylation of Lys4 of histone H3; H3K4me3, tri-methylation of Lys4 of histone H3; UBF, upstream binding factor; IGS, non-coding intergenic spacer; TCP, Tranylcypromine
