# Supplementary figures and images for: TBX1 Functions as a Tumor Activator in Prostate Cancer by Promoting Ribosome RNA Gene Transcription
Source: Front Oncol. 2021 Jan 26;10:616173. doi: 10.3389/fonc.2020.616173 (PMC7871003; doi:10.3389/fonc.2020.616173)

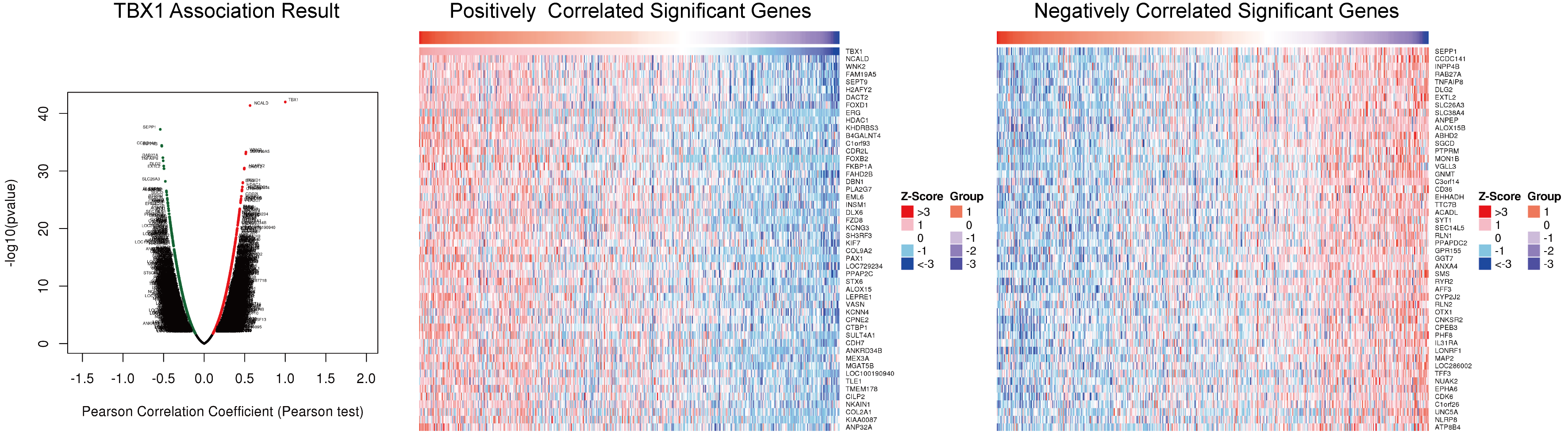

Supplement: Supplemental Figure 1 — Genes differentially expressed in correlation with TBX1 in PCa (LinkedOmics). Left, the correlations between TBX1 and genes differentially expressed was analyzed using the Pearson test; Middle, the TOP 50 genes positively correlated with TBX1 were shown using the heat map; Right, the TOP 50 genes negatively correlated with TBX1 were shown using the heat map. The red color represents positively correlated genes and the green color represents negatively correlated genes. Prostate cancer, Pca. [file Image_1.tif]
