# Supplementary material for: TBX1 Functions as a Tumor Activator in Prostate Cancer by Promoting Ribosome RNA Gene Transcription
Source: Front Oncol. 2021 Jan 26;10:616173. doi: 10.3389/fonc.2020.616173 (PMC7871003; doi:10.3389/fonc.2020.616173)
Supplement: Supplementary file 2 [file DataSheet_1.docx]

**Supplemental Table1** The primers used for q-PCR

| **Primer** | **Type** |  | **Sequence 5’-3’** |
| --- | --- | --- | --- |
| TBX1 | forward |  | ACGACAACGGCCACATTATTC |
|  | reverse |  | CCTCGGCATATTTCTCGCTATCT |
| 45SrRNA | forward |  | GAACGGTGGTGTGTCGTT |
|  | reverse |  | GCGTCTCGTCTCGTCTCACT |

**Supplemental Table 2** The rDNA primers used for ChIP assay

| **Primer** | **Type** |  | **Sequence 5’-3’** |
| --- | --- | --- | --- |
| *H42* | forward |  | GCTTCTCGACTCACGGTTTC |
|  | reverse |  | CCGAGAGCACGATCTCAAA |
| *H42.9* | forward |  | CCCGGGGGAGGTATATCTTT |
|  | reverse |  | CCAACCTCTCCGACGACA |
| *H4* | forward |  | CGACGACCCATTCGAACGTCT |
|  | reverse |  | CTCTCCGGAATCGAACCCTGA |
| *H8* | forward |  | AGTCGGGTTGCTTGGGAATGC |
|  | reverse |  | CCCTTACGGTACTTGTTGACT |
| *H13* | forward |  | ACCTGGCGCTAAACCATTCGT |
|  | reverse |  | GGACAAACCCTTGTGTCGAGG |
| *H18* | forward |  | GTTGACGTACAGGGTGGACTG |
|  | reverse |  | GGAAGTTGTCTTCACGCCTGA |
| *H27* | forward |  | CCTTCCACGAGAGTGAGAAGCG |
|  | reverse |  | CTCGACCTCCCGAAATCGTACA |
| *H32* | forward |  | GGAGTGCGATGGTGTGATCT |
|  | reverse |  | TAAAGATTAGCTGGGCGTGG |
